# Raccoon Dogs Adjust Diel Visitation at Scent Marking Latrines to Reduce Human Disturbance in Urban Areas

**DOI:** 10.1002/ece3.72695

**Published:** 2025-12-17

**Authors:** Shigeru Osugi, Maximilian L. Allen, Shinsuke Koike

**Affiliations:** ^1^ United Graduate School of Agricultural Science Tokyo University of Agriculture and Technology Tokyo Japan; ^2^ Illinois Natural History Survey University of Illinois Champaign Illinois USA; ^3^ Institute of Global Innovation Research Tokyo University of Agriculture and Technology Tokyo Japan

**Keywords:** activity pattern, anthropogenic, behavioral flexibility, diel activity, *Nyctereutes procyonoides*, omnivore

## Abstract

Human activity and development have a variety of effects on wildlife behavior, often prompting urban wildlife to adopt behavioral strategies—including spatiotemporal activity adjustments—that facilitate persistence in human‐dominated environments. In this study, our objective was to determine whether and how visitation behavior of raccoon dogs *(Nyctereutes procyonoides)* to communal scent marking hubs (i.e., latrines) was influenced by human activity and development in urban green spaces in Tokyo, Japan. Using camera‐trap data collected over 4530 trap days, we recorded 3259 latrine visits and assessed temporal patterns of activity at both the green space and individual latrine scales. We found that raccoon dog visitation to latrines was primarily at night in the urban green spaces, but the peak times for visitation at each latrine varied depending on the environmental context of the green space (size and the surrounding environment) and the latrines (the distance from a road). Our findings indicate that while raccoon dogs maintain scent‐marking behavior in urban areas, they exhibit behavioral flexibility through adjusting the timing of visitation to minimize disturbance from human activity. Our results underscore the importance of considering the spatiotemporal behavioral adaptations of urban wildlife and highlight the need to preserve accessible and temporally buffered areas for olfactory communication in human‐dominated areas.

## Graphical abstract

The diurnal pattern of latrine visits by raccoon dogs for each study site‐year based on kernel density estimates. Dashed vertical lines indicate the annual mean times of sunset and sunrise, and dotted vertical lines indicate the mean times at 21:00, 0:00, and 3:00.

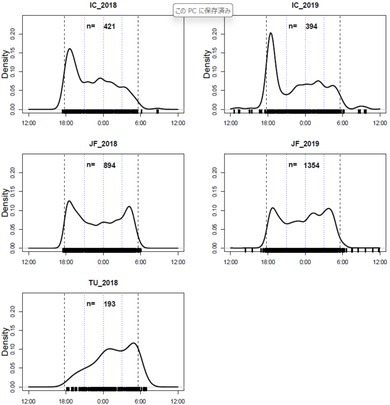

## 1 Introduction

Human activities have a variety of effects on wildlife behavior, especially in urban areas (Ditchkoff et al. 2006; Sol et al. 2013). Urban‐adapted species often exhibit a range of behavioral adjustments that facilitate their success in human‐dominated environments (Lowry et al. 2013; Sol et al. 2013). These adjustments often include spatiotemporal avoidance and a reduction in their fear of humans (Sol et al. 2013; Weiss et al. 2023). For instance, urban mammals often display shorter flight initiation distances compared to those in rural areas (e.g., Mayer et al. 2025)—an indicator of increased tolerance to human presence. Yet urban terrestrial mammals usually face greater constraints in spatially avoiding humans and consequently tend to instead respond through temporal avoidance (Gaynor et al. 2018). A common strategy used by urban mammals to minimize human disturbance is altering their activity patterns to be crepuscular or nocturnal so that they avoid periods when humans are most active (Gaynor et al. 2018). For example, species such as European hares (*Lepus europaeus Pallas*), red foxes ( *Vulpes vulpes* ), coyotes ( *Canis latrans* ), and bobcats ( *Lynx rufus* ) may shift their activity to twilight or even become nocturnal due to human activity being more diurnal (Kitchen et al. 2000; Tigas et al. 2002; Riley et al. 2003; Gil‐Fernández et al. 2020; Krivopalova et al. 2024; Allen et al. 2025). But wildlife varies in their responses to human activity based on different behaviors, with scent marking and denning behaviors often being most sensitive (Wilmers et al. 2013; Yovovich et al. 2020).

Many mammals use scent marking as a form of chemical communication for the advertisement of territorial use and mate attraction (Gosling and Roberts 2001; Allen et al. 2016). These olfactory signals are typically deposited in strategic locations with high visibility and longevity for conspecifics to find (Vogt et al. 2014; Allen et al. 2017; Krofel et al. 2025). However, in urban areas, the signals from scent marking may be disturbed or destroyed by human activity, potentially leading animals to scent mark in less preferred locations in order to not be disturbed or shift to using scent marking sites at times when they are less likely to be disturbed. Human disturbance thus has the potential to negatively affect the social structure, reproduction, and spatial dynamics of urban mammal populations, but this aspect of scent marking has been understudied.

The raccoon dog ( *Nyctereutes procyonoides* ) is an omnivorous mesocarnivore (body length = 68–81 cm, weight = 3.2–5.0 kg) that is widely distributed throughout the forested areas of the temperate regions of East Asia. In Japan, there are two subspecies of raccoon dogs: *Nyctereutes p*. *albus* inhabits Hokkaido Island, whereas *Nyctereutes p. viverrinus* inhabits Honshu, Shikoku, and Kyushu Islands. Raccoon dogs in Japan inhabit a wide range of habitats, from coastal to subalpine zones throughout the country, and inhabit not only mountainous areas but also farmland, urban areas, and other areas close to human habitation (Ohdachi et al. 2009; Soga and Koike 2013). Raccoon dogs are opportunistic omnivores and mainly eat fruit, invertebrates, and small vertebrates (Ohdachi et al. 2009). Some raccoon dogs inhabiting urban peripheries also depend on human‐provided food such as leftovers and trash. However, there are some areas in central Tokyo that are completely independent of artificial food, and even within urban areas, the degree of dependence on artificial food varies by area (e.g., Akihito et al. 2016; Takatsuki and Kobayashi 2023). Furthermore, in urban areas that are unaffected by artificial food, there is no significant difference in dietary habits between urban and rural areas (Akihito et al. 2016). Similar to other urban mammals, raccoon dogs show a nocturnal shift in their foraging behavior for fallen fruit in urban forests compared to mountain forests (Osugi, Trentin, and Koike 2022). Additionally, in a period without human interference during the COVID‐19 lockdown in 2020, raccoon dogs showed substantial shifts to be more diurnal in their activity patterns (Osugi, Baek, et al. 2022). Furthermore, although raccoon dogs are hunted in Japan, capturing them with guns is prohibited around urban areas, and no hunting with traps has been reported in the study area. This suggests that raccoon dogs adjust their foraging activity in response to their fear of just human presence, but it is unknown if human disturbance affects their scent‐marking behavior.

Raccoon dog scent marking typically consists of habitually defecating in communal latrines that act as a social hub for communication among individuals in the population (Ikeda 1984; Yamamoto 1984). Latrines are typically in flat, wooded areas (Ikeda 1984) or on ridges (Watanabe et al. 2021), but scent marking activity and latrine locations can shift seasonally (Ikeda 1984; Watanabe et al. 2021). Raccoon dog latrines are also often used by other mammals (Yoshida and Saito 2022), but there is limited information on latrine use by raccoon dogs in urban areas or how it may be affected by human disturbance. The one previous urban study of raccoon dog latrine use found that in green spaces in the center of Tokyo, raccoon dogs avoid human disturbance by changing their temporal—but not spatial—activity patterns (Tsunoda et al. 2019). However, although this green space is vast (51 ha), it is characterized by strict restrictions on public access during the day and night (the Akasaka Imperial Grounds: AIG), which may reduce the generalizability of those findings to other urban green spaces with normal or higher levels of human activity.

In this study, we investigated the impact of human activities on the scent marking behavior of urban raccoon dogs by assessing the activity patterns at raccoon dog latrines in a commuter town near Tokyo, Japan. Specifically, we tested the following hypotheses: (1) raccoon dogs predominately visit latrines for scent marking during nocturnal hours to avoid being disturbed by humans, similar to their foraging behavior previously observed at the same site (Osugi, Trentin, and Koike 2022). (2) The diel activity patterns while using latrines will vary by latrine location based on two spatial scales: (2a) at the large scale (equivalent to a raccoon dog's home range—several 10 ha; e.g., Mise et al. 2016), the activity patterns at latrines vary depending on the overall size and quality of the green space, as well as the environment surrounding the green space; (2b) at the small scale (the immediate area around a latrine—i.e., within 100 m), the activity patterns at latrines vary depending on how the individual location of the latrines is affected by human disturbance, especially how far they occur from the road.

## 2 Material and Methods

### 2.1 Study Area

We conducted our study at three green spaces within 10 km of each other in western Tokyo (35°40–41° N, 139°28–33° E). The three study sites were the International Christian University campus (hereafter IC: 65 ha, 35°41′ N, 139°28′ E, two latrines, number of students and staff: about 3700), the Tokyo Metropolitan Nogyo High School, Jindai Farm (hereafter JF: 2.5 ha, 35°40′ N, 139°33′ E, three latrines, number of students and staff: about 300), and the Tokyo University of Agriculture and Technology, Fuchu campus (hereafter TU: 43 ha, 35°41′ N, 139°29′ E, three latrines, number of students and staff: about 2500). These study sites are situated in commuter towns in western Tokyo, with population densities of approximately 11,900 people per square kilometer in Mitaka City (IC), approximately 11,200 people per square kilometer in Chofu City (JF), and approximately 8900 people per square kilometer in Fuchu City (TU). Although there is no information available on the level of human activity at the study sites and their surrounding areas by time of day, it is expected that human activity at university and school facilities is significantly higher during the day than at night.

Most of IC is forest cover dominated by deciduous and evergreen broadleaf trees such as *Quercus serrata* , *Q. acutissima* , *Carpinus tschonoskii*, *Q. myrsinifolia* , and *Cornus controversa* . To the south of IC is a neighboring large park (40 ha), and the rest of the area is surrounded by residential areas. JF is a long and narrow valley, dominated by deciduous broadleaf trees such as *Quercus serrata* and farmland, and bordered by residential areas and some parks. TU has patchy small forests (such as *Zelkova serrata* and *Cercidiphyllum japonicum* ) surrounded by residential areas.

### 2.2 Latrine Visit Record

Before the survey began, in February and March 2018, we walked around the study site and found the latrines based on information we obtained from staff and local residents. We then installed cameras between April 2018 and March 2020 except TU (from April 2018 to March 2018) (Table 1). We identified two latrines in IC, three in IF, and three in TU, respectively, and installed a total of eight cameras, one at each latrine.

**TABLE 1:** The information of target latrines and survey periods, number of defecation times, percent of night visit, and camera trap days. The black bars indicate periods when raccoon dogs were recorded using the latrines, and the white bars indicate periods when raccoon dogs were not recorded using the latrines.

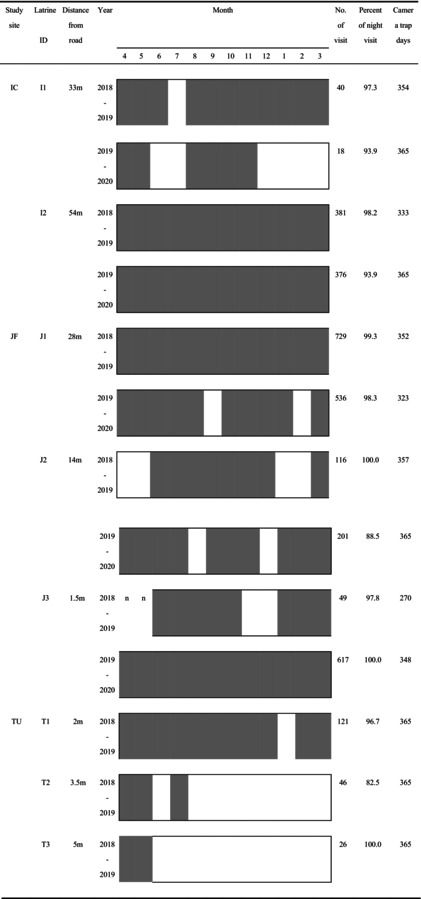

At the start of each annual survey period, we mapped all active raccoon dog latrines in each study site. To record the behavior of raccoon dogs at each latrine, we installed a camera trap (Ltl Acorn Ltl‐6210MC, Zhuhai Ltl Acorn Electronics Co. Ltd., Zhuhai, China) at a height of 40–60 cm on a tree trunk that was 1–3 m away from the latrines. We programmed the camera traps to take videos for 20s with a 0 s delay between activity. From the recorded videos, we documented the date, time, and latrine ID of raccoon dogs that defecated at each latrine. If a further video clip was recorded within 60 s of the first event, we categorized both clips as 1 event.

### 2.3 Statistical Analyses

To analyze the diel activity of raccoon dogs during visits to latrines, we estimated the activity patterns for each study site and each latrine using kernel density estimation with the overlap package (Meredith and Ridout 2021) based on the time of each visit to the latrines. We used solartime from the “activity” package to correct for seasonal differences between daytime and nighttime periods relative to the sunrise and sunset times (Vazquez et al. 2019). When comparing the activity pattern of latrine visits between study sites, we pooled data from all latrines located in that same study site for each year. At that time, we compared each set of study sites‐years for when there was a total of 120 or more visits to latrines in that year (Table 1). When comparing the activity pattern of latrine visits between each latrine, we compared each latrine‐year in years when there was a total of 120 or more visits to each latrine in that year (Table 1).

To compare the activity patterns of latrine visits between each study site‐year and between each latrine‐year, we calculated the overlap coefficient (Δ, ranging from 0 [no overlap] to 1 [complete overlap]). Regarding the overlap coefficient, according to the evaluation criteria (Monterroso et al. 2014), the overlap coefficient (Δ) is **≤** 0.5as “low,” 0.5 < to ≤ 0.75 as “medium,” and 0.75 or more as “high.” Following the criteria set out by Meredith and Ridout (2021), we used the Δ_1_ estimator when the smaller of the sample sizes was less than 50 visits, and the Δ_4_ estimator when the smaller of the sample sizes was 50 or more visits. To assess the reliability of these estimated Δ statistics and estimate the 95% confidence intervals (CI), we performed a smoothed bootstrap with 1000 bootstrap samples. We assessed the statistical significance of the differences in the diurnal pattern of latrine visits between each latrine and between each latrine‐year, using Watson's two‐sample test (Pewsey et al. 2013). We performed all analyses in the program R (ver. 4.2.1; R Core Team 2022), and we considered *p* < 0.05 to be statistically significant in each analysis.

## 3 Results

We documented a total of 3259 (mean ± SD, 250.7 ± 249.3 per each latrine‐year) latrine visits over a total of 4530 trap days (mean ± SD, 348.5 ± 27.2 days per each latrine‐year). Activity was primarily during the nighttime (defined as the period from sunset to sunrise), with 93.8%–99.3% (mean ± SD, 96.6% ± 2.5%) (Table 1) of visits to latrines during the nighttime for each study site‐year.

**FIGURE 1:**
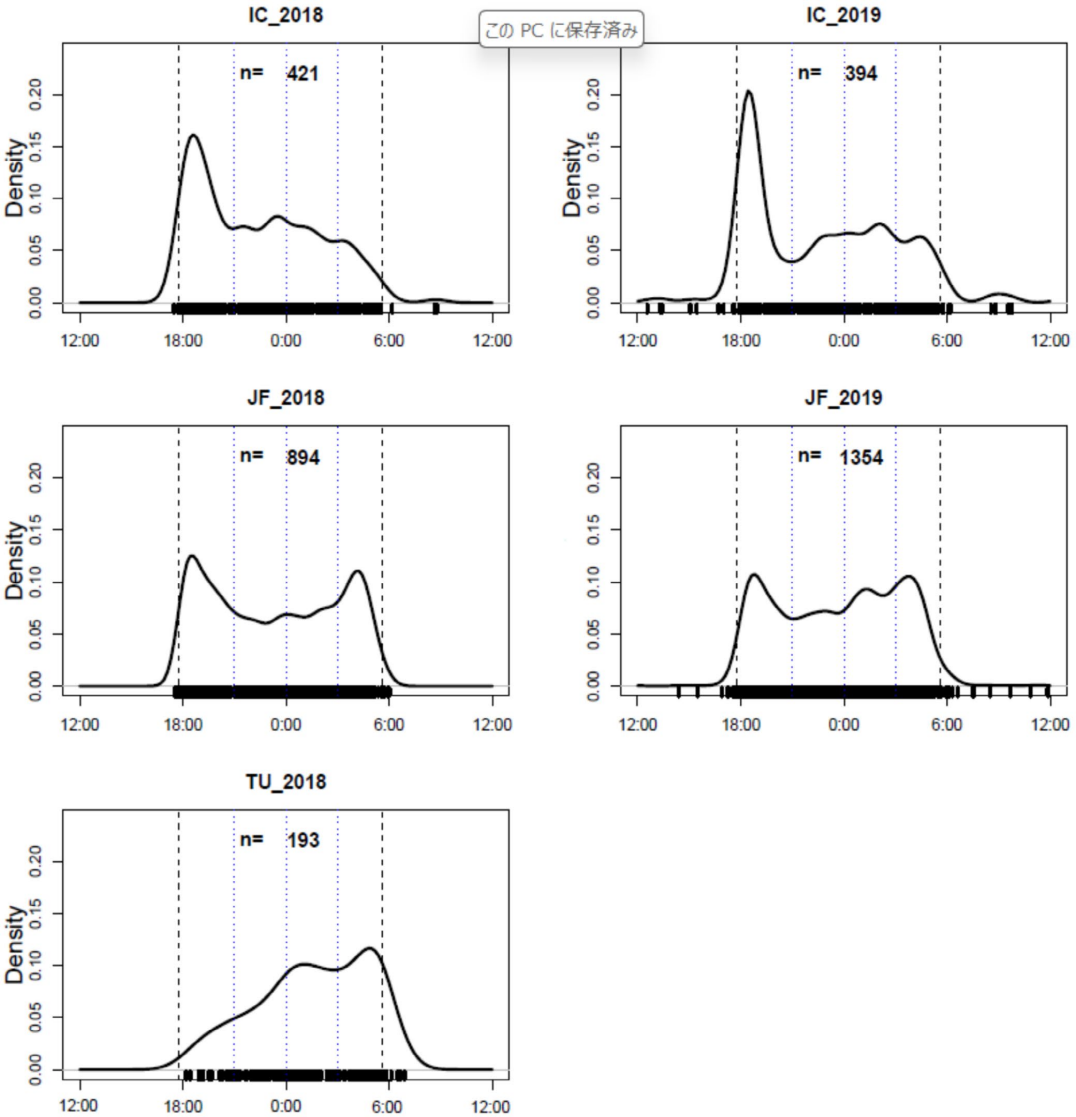
Diurnal pattern of latrines' visit by raccoon dogs for each study site‐year based on kernel density estimates. Dashed vertical lines indicate the annual mean times of sunset and sunrise, and dotted vertical lines indicate the mean times at 21:00, 0:00, and 3:00.

To evaluate differences in the diel pattern of latrine visits between study sites‐years, we compared five study sites‐years (Table 2). We found significant differences for all study sites‐years combinations (Table 2). Despite high temporal overlap, the diurnal patterns of toilet usage differ due to their peak times occurring at different hours. The overlap coefficient (Δ) between IC and JF was high, and the peak times of latrine visits were just after sunset for IC and just after sunset and just before sunrise for JF. On the other hand, in TU, no peak was observed just after sunset, but a peak was observed just before sunrise (Figure 1).

**TABLE 2:** Overlap coefficient of diurnal pattern of latrines' visit between study sites–years.

Study site_year	IC_2018	IC_2019	JF_2018	JF_2019
IC_2019	0.861*** (0.809–0.906)			
JF_2018	0.868*** (0.827–0.909)	0.830*** (0.783–0.875)		
JF_2019	0.836*** (0.789–0.880)	0.794*** (0.743–0.847)	0.916*** (0.885–0.952)	
TU_2018	0.643*** (0.575–0.705)	0.657*** (0.597–0.717)	0.746*** (0.690–0.799)	0.791*** (0.738–0.840)

*Note:* The values in parenthesis indicate 95% confidence interval. Low overlap, moderate overlap, and high overlap indicate that *D* values were *D* ≤ 0.50, 0.50 < *D* ≤ 0.75, and *D* > 0.75, respectively, bold indicates high overlaps. Test results by Watson's two‐sample test: ***p < 0.001, **p < 0.01, *p < 0.05.

**FIGURE 2:**
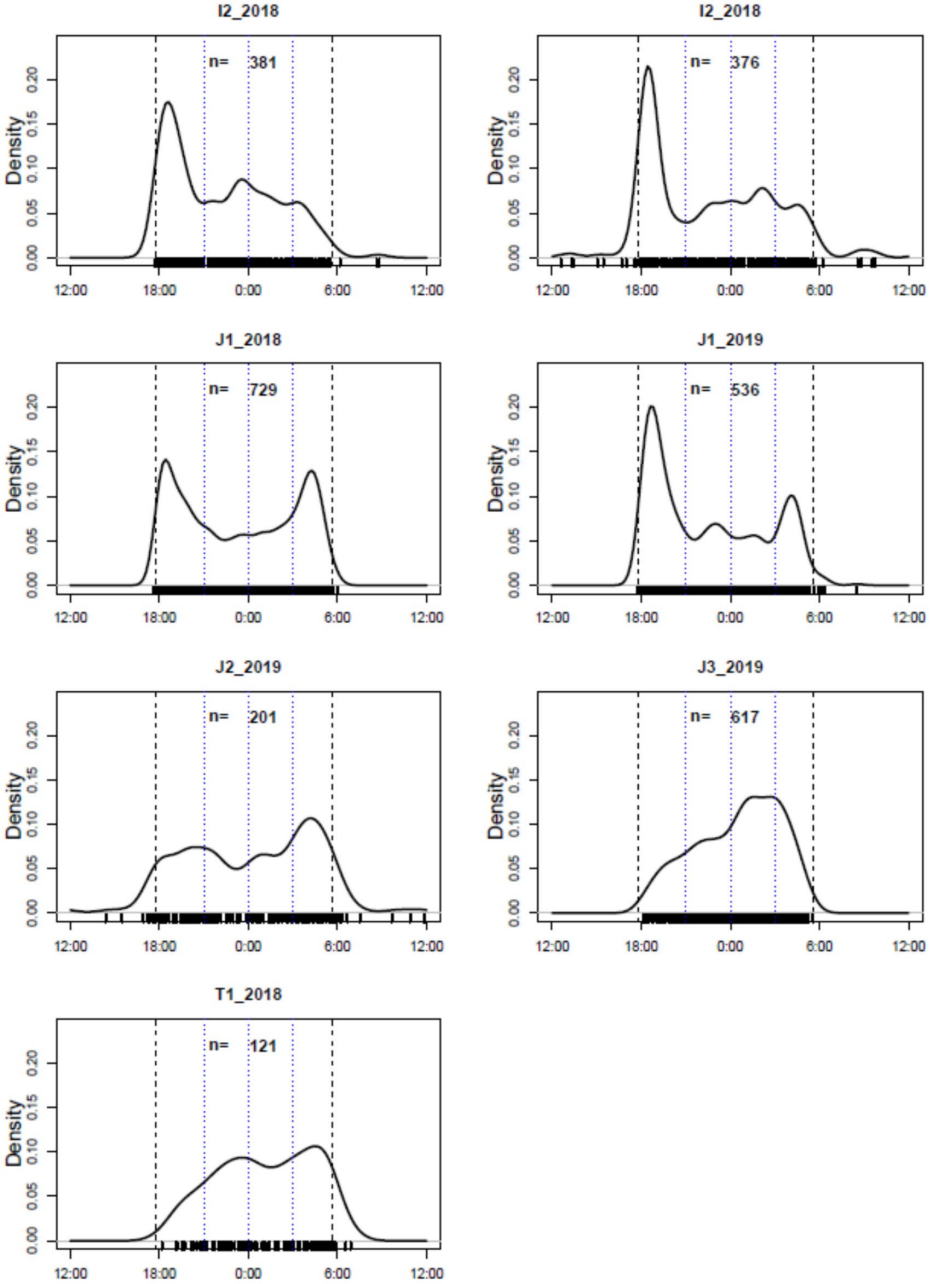
Diurnal pattern of latrine visits by raccoon dogs for each latrine‐year based on kernel density estimates. Dashed vertical lines indicate the annual mean times of sunset and sunrise, and dotted vertical lines indicate the mean times at 21:00, 0:00, and 3:00.

We further evaluated the diel pattern of latrine visits between each of seven latrine‐year combinations (Table 3). We found significant differences except for the combination of J1 in 2018 and J2 in 2019 (Δ = 0.89) (Table 3). There were 11 combinations with a high overlap coefficient between latrine‐years, 10 combinations with a medium overlap coefficient, and no combinations with a low overlap coefficient (Table 3, Figure 2, Appendix S1). The peak times of latrine visit, I2_2018 and I2_2019 and T1_2018 showed the same trend as in the study site, but J2_2019 and J3_2019 showed patterns different from those in the study site. In J2_2019, the peak time for latrine visit was just after sunrise, whereas in J3_2019, similar to T1_2018, no peak was observed just after sunset (Figure 2).

**TABLE 3:** Coefficient of overlaps of temporal latrine visit patterns.

Latrine ID_year	I2_2018	I2_2019	J1_2018	J1_2019	J2_2019	J3_2019
I2_2019	0.865*** (0.815–0.915)					
J1_2018	0.839** (0.793–0.883)	0.813*** (0.767–0.860)				
J1_2019	**0.889** (0.836–0.939)	0.854*** (0.805–0.896)	0.878*** (0.826–0.924)			
J2_2019	0.735*** (0.664–0.802)	0.734*** (0.670–0.797)	0.858* (0.803–0.903)	0.762*** (0.701–0.820)		
J3_2019	0.693*** (0.638–0.746)	0.640*** (0.587–0.693)	0.715*** (0.671–0.760)	0.674*** (0.627–0.719)	0.744*** (0.680–0.808)	
T1_2018	0.680*** (0.609–0.748)	0.650*** (0.584–0.716)	0.751*** (0.690–0.814)	0.673*** (0.609–0.741)	0.836** (0.765–0.904)	0.849** (0.779–0.909)

*Note:* The values in parenthesis indicate 95% confidence interval. Low overlap, moderate overlap, and high overlap indicate that *D* values were *D* ≤ 0.50, 0.50 < *D* ≤ 0.75, and *D* > 0.75, respectively, the bold indicates high overlaps. Test results by Watson's two‐sample test: ***p < 0.001, **p < 0.01, *p < 0.05.

## 4 Discussion

In our study of latrine site visitation in urban green spaces in Tokyo, we found support for our hypothesis that raccoon dogs would be primarily nocturnal and partial support for our hypothesis that visitation would vary among latrine sites. Previous studies conducted in forested areas with low human activity indicate raccoon dogs are nocturnal (Ikeda et al. 2016; Watabe and Saito 2022; Wang et al. 2024; Wooldridge et al. 2024), although a certain proportion of daytime activity has been reported (daytime activity 15.1% (Ikeda et al. 2016), 17.6% (Jasiulionis et al. 2023)). Regarding visits to latrines, while 36.7% of visits occurred during the day in AIG areas with low human activity (Tsunoda et al. 2019), this study found only 4.4% of visits during the day, indicating they are primarily nocturnal. Many urban animals shift to more nocturnal activity to reduce their interaction with humans (Gaynor et al. 2018), which may be the reason that latrine visitation by raccoon dogs is primarily nocturnal. But certain latrines diverged from site‐wide patterns, suggesting that fine‐scale environmental factors (e.g., visibility, proximity to roads, refuge size) may influence the timing of visitation to latrines. Raccoon dogs are known to locate latrines near feeding sites and travel corridors (Ikeda 1984; Watanabe et al. 2021). In this study, some of the latrines were also located near roads with heavy pedestrian and vehicle traffic, and near residential areas that functioned as animal feeding grounds at night. Although such places seem suboptimal due to the increased human activity, raccoon dogs often use them when human activity is low. This suggests that even in developed areas, latrines perform important functions for intraspecific olfactory communication (Gosling 1982; Ikeda 1984; I. Yamamoto 1984).

Comparisons with previous work illustrate the behavioral plasticity of raccoon dogs and how human activity affects their latrine visitation behavior. In our study site—where the survey site consists of university and high school facilities where human activity is high during the day and low at night. Although roads are active at all times due to the surrounding residential areas, there is almost no activity in the middle of the night, and the percentage of nighttime latrine visits by raccoon dogs ranged from 82.5% to 100%, supporting hypothesis 1. This is different from the one previous study on latrine visitation by raccoon dogs in urban areas in green space (AIG) where public access was restricted (Tsunoda et al. 2019)—which found that although latrine visits were 63.3% at night, many visits were also observed during the day. The less nocturnal activity in those areas suggests that strict restrictions on public access led to a reduced need for nocturnal avoidance of humans. This pattern suggests that raccoon dogs have behavioral plasticity in their scent marking behavior, similar to the pattern in raccoon dogs when foraging (Osugi, Trentin, and Koike 2022).

Site‐level differences in diel patterns showed some support for hypothesis 2—that environmental context shapes raccoon dog latrine activity. Although the three green spaces we used as study sites were all protected, their environments and surrounding areas differed and may have influenced the diel activity of raccoon dogs at latrines between green spaces. A previous study at AIG showed that raccoon dogs stay within AIG throughout the whole day and use it for resting and feeding (Mitsuhashi et al. 2018). The diel pattern of latrine visits in the AIG occurred primarily at sunset, with visits at night and almost no visits at sunrise (Tsunoda et al. 2019). A similar diurnal pattern was observed at IC—which has a large green space including a large forest area—so raccoon dogs may complete their daily activities within the site similar to AIG. On the other hand, JF showed peaks in visits after sunset and before sunrise, while TU showed no peaks after sunset at all, with visitation increasing toward the middle of the night. Although JF is small in area, there are forests inside and some parks around it; TU is large in area; however, there are limited forests inside, and it is surrounded by residential areas. Raccoon dogs inhabiting isolated green spaces in urban areas are known to rest in the green spaces during the day and move to surrounding residential areas and green spaces at nighttime to feed (Y. Yamamoto 1993). There is no information on the behavior of raccoon dogs in this study area so far, but it is possible that the daily activities of raccoon dogs are not completed within the JF or TU and that they simply use the JF or TU as a migration route at night. The timing of visits to latrines may have differed due to these differences since the diel pattern of latrine visits in urban green spaces differs depending on the quality of the green space and the surrounding environment.

We also observed within‐site variation among latrines, showing possible support for hypothesis 2b. For example, in JF, where multiple latrines were observed in the same year, J2 and J3 showed usage patterns that were different from those of the entire study site. Specifically, whole JF had peaks after sunset and before sunrise, J2 only had peaks just before sunrise, and J3 had no peaks just after sunset or just before sunrise. The biggest difference between the environments among these latrines in JF was the distance from the road. J1 was 28 m and J2 was 14 m away from the road, while J3 was only a few meters away, and the diel pattern was similar to that of T1, which was also adjacent to a road. In general, road traffic noise is attenuated as the distance from the road increases, and wild animals become wary of road traffic noise and become less active depending on the intensity of the sound (Shannon et al. 2014). It is possible that in J3, which is adjacent to the road, they avoided visiting the raccoon dog latrines in the early hours after sunset when traffic volume is still high. These findings suggest that site‐level conditions can modulate the timing of activity at latrines, but further verification is needed.

Latrines and other scent marking sites have important roles in the ecological and social functioning of mammal communities (Gosling and Roberts 2001; Allen et al. 2016). The consistent use of latrines across sites by raccoon dogs, despite human presence, suggests the importance of latrines for olfactory communication. The variability in timing implies behavioral flexibility—both among green spaces and individual sites—likely to optimize communication while balancing risk. Our study only focused on urban sites, limiting our ability to disentangle urbanization effects from natural behavioral variability. Future studies could compare urban and rural scent marking activity and incorporate additional measures of human activity (e.g., acoustic measurements for traffic or human visitation counters) to understand the direct effects of human activity on marking activity. These findings are an important step in understanding how scent‐based communication is maintained in urbanized settings and have implications for urban planning and conservation—specifically, maintaining temporal refuges and access to sites that can serve as scent marking areas may support urban mammal persistence.

## Author Contributions

**Shigeru Osugi:** conceptualization (equal), data curation (lead), formal analysis (lead), investigation (lead), methodology (lead), software (lead), visualization (lead), writing – original draft (lead). **Maximilian L. Allen:** conceptualization (equal), supervision (supporting), validation (supporting), writing – review and editing (equal). **Shinsuke Koike:** conceptualization (equal), data curation (supporting), formal analysis (supporting), funding acquisition (lead), investigation (supporting), methodology (supporting), project administration (lead), software (supporting), supervision (lead), validation (lead), writing – original draft (supporting), writing – review and editing (equal).

## Funding

This work was supported by Japan Society for the Promotion of Science (JP24H01429), Institute of Global Innovation Research, and Tokyo University of Agriculture and Technology.

## Conflicts of Interest

The authors declare no conflicts of interest.

## Supporting information


**Data S1:** ece372695‐sup‐0001‐DataS1.xlsx.

**Appendix S1:** ece372695‐sup‐0002‐AppendixS1.docx.

## Data Availability

The data analyzed in the manuscript is available as Supporting Information.
