# Supplementary material for: Raccoon Dogs Adjust Diel Visitation at Scent Marking Latrines to Reduce Human Disturbance in Urban Areas
Source: Ecol Evol. 2025 Dec 17;15(12):e72695. doi: 10.1002/ece3.72695 (PMC12709652; doi:10.1002/ece3.72695)

Appendix S1. Temporal overlap of diurnal patterns of latrine visit by kernel density estimates between each latrine-years. Grey shared areas indicate the coefficient of overlaps of the two density estimates. Dashed vertical lines indicate mean times of sunset and sunrise, and dotted vertical lines indicate the mean times at 21:00, 0:00, and 3:00.


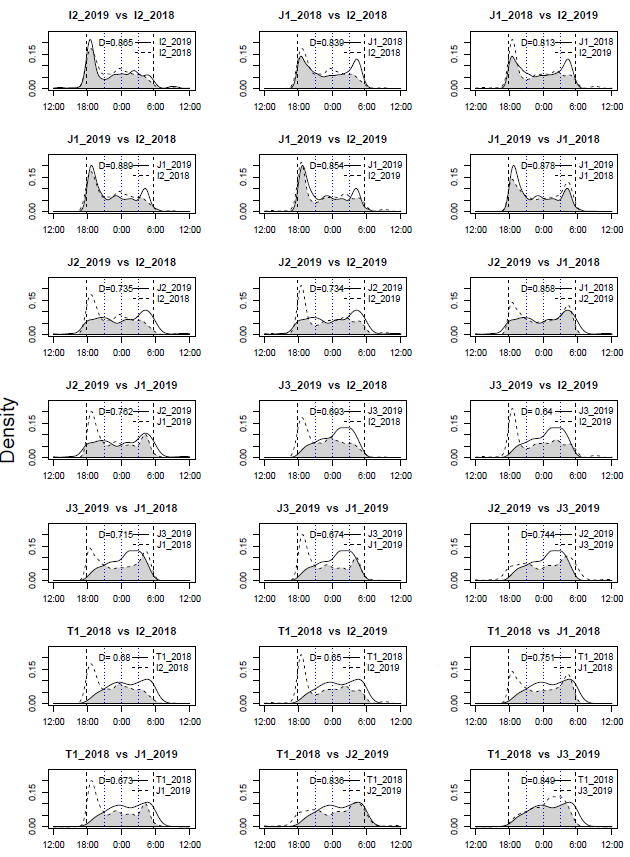

Supplement: Supplementary file 2 — Appendix S1: ece372695‐sup‐0002‐AppendixS1.docx. [file ECE3-15-e72695-s001.docx]
